# Supplementary material for: Drug repurposing for aging research using model organisms
Source: Aging Cell. 2017 Jun 16;16(5):1006–15. doi: 10.1111/acel.12626 (PMC5595691; doi:10.1111/acel.12626)
Supplement: Supplementary file 7 — Data S1 Zip‐Archive of all report cards. [file ACEL-16-1006-s007.zip › RC_8ST.pdf]

8ST

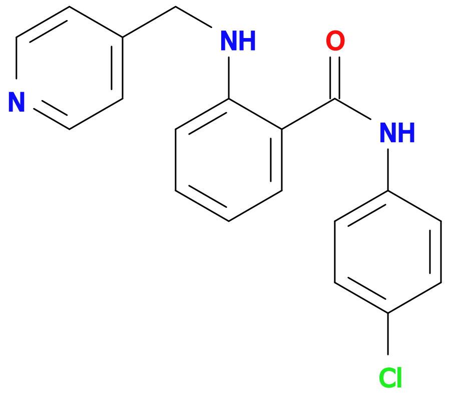

Database identifiers

ChEMBLCompound	CHEMBL101683
DrugBank	DB07288
ZINC	ZINC00008732
eMolecules	28183501

Ranking

	Rank	Score
Drosophila	432/697	0.331
C. elegans	NA	NA

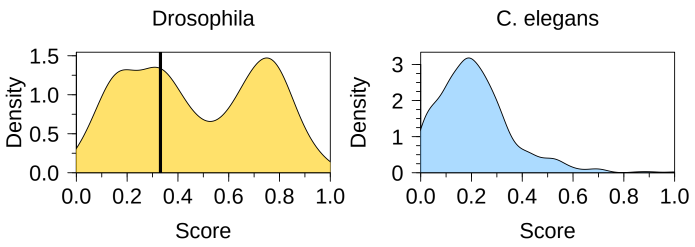

	Ageing implication	Domain conservation	Binding site conservation	Binding affinity	Bioavailability	Lipinski	Promiscuity	Purchasability	Drug approval	**Total**
Drosophila	0.203	0.929	0.99	0.934	(0.9)	0.0	-0.0	0.1	0.075	0.331
C. elegans	NA	NA	NA	NA	NA	NA	NA	NA	NA	NA

Names

No synonyms found

Roles

ChEBI entry None has no roles

Status

Approved drug (according to ChEMBL)	No
Classification (according to DrugBank)	experimental
Number of Rule of 5 violations	0
Binding affinity to original target in log units (RF-Score prediction)	7.66
Burns *C. elegans* bioavailability prediction	6.78

Compound Target Characteristics

Vascular endothelial growth factor receptor 1

Best gene implication in ageing for this target family came from gene P17948 via mapping the annotation from Ensembl ENSG00000102755 via mapping the annotation from EntrezGene 2321 via mapping the annotation from GenAgeHuman 0171 annotated in GenAge release 17.

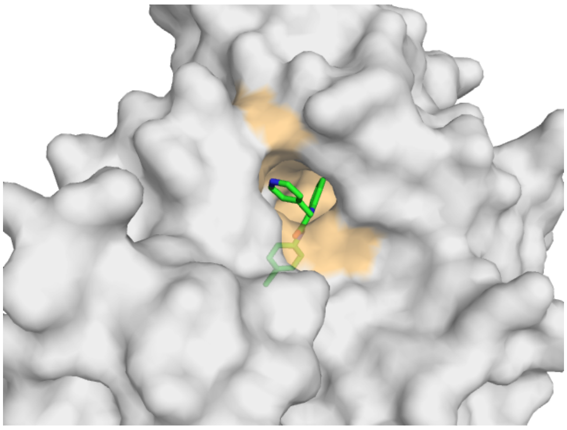

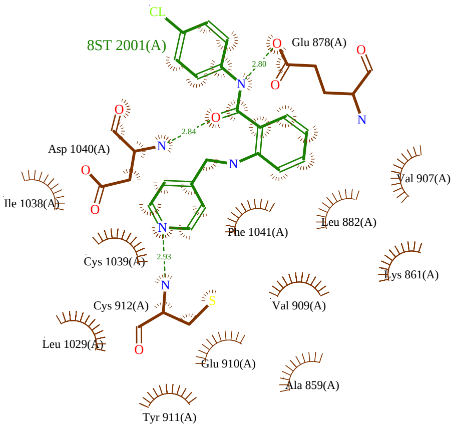

protein	amino acids contacts (binding site)														
PDB:3hng:chainA:P17948	A	K	E	L	V	V	E	Y	C	L	L	I	C	D	F
sp:P17948:VGFR1_HUMAN	A	K	E	L	V	V	E	Y	C	L	L	I	C	D	F
tr:L7RSL3:L7RSL3_HUMAN	A	K	E	L	V	V	E	Y	C	L	L	I	C	D	F
tr:G3V633:G3V633_RAT	A	K	E	L	V	V	E	Y	C	L	L	I	C	D	F
sp:P35969:VGFR1_MOUSE	A	K	E	L	V	V	E	Y	C	L	L	I	C	D	F
tr:C1C3C7:C1C3C7_DROME	A	K	E	M	V	V	E	Y	C	L	L	I	C	D	F
tr:B6IDV6:B6IDV6_DROME	A	K	E	M	V	V	E	Y	C	L	L	I	C	D	F
tr:Q8T9F6:Q8T9F6_DROME	A	K	E	M	V	V	E	Y	C	L	L	I	C	D	F
tr:E1JHB8:E1JHB8_DROME	A	K	E	M	V	V	E	Y	C	L	L	I	C	D	F
tr:Q8T4M0:Q8T4M0_DROME	A	K	E	M	V	V	E	Y	C	L	L	I	C	D	F
tr:Q7KTI7:Q7KTI7_DROME	A	K	E	M	V	V	E	Y	C	L	L	I	C	D	F
tr:Q8T4L8:Q8T4L8_DROME	A	K	E	M	V	V	E	Y	C	L	L	I	C	D	F
tr:Q9VLQ8:Q9VLQ8_DROME	A	K	E	M	V	V	E	Y	C	L	L	I	C	D	F
tr:Q8IPG1:Q8IPG1_DROME	A	K	E	M	V	V	E	Y	C	L	L	I	C	D	F
tr:Q7KTI8:Q7KTI8_DROME	A	K	E	M	V	V	E	Y	C	L	L	I	C	D	F
tr:Q95P10:Q95P10_DROME	A	K	E	M	V	V	E	Y	C	L	L	I	C	D	F
tr:E1JHB6:E1JHB6_DROME	A	K	E	M	V	V	E	Y	C	L	L	I	C	D	F
tr:E1JHB7:E1JHB7_DROME	A	K	E	M	V	V	E	Y	C	L	L	I	C	D	F

protein	whole protein ident	simil	domain-based ident	simil	contact-based ident	simil
PDB:3hng:chainA:P17948	1.0	1.0	1.0	1.0	1.0	1.0
sp:P17948:VGFR1_HUMAN	1.0	1.0	1.0	1.0	1.0	1.0
tr:L7RSL3:L7RSL3_HUMAN	1.0	1.0	1.0	1.0	1.0	1.0
tr:G3V633:G3V633_RAT	0.82	0.95	0.96	0.99	1.0	1.0
sp:P35969:VGFR1_MOUSE	0.81	0.94	0.96	0.99	1.0	1.0
tr:C1C3C7:C1C3C7_DROME	0.17	0.5	0.54	0.86	0.93	0.99
tr:B6IDV6:B6IDV6_DROME	0.2	0.6	0.54	0.86	0.93	0.99
tr:Q8T9F6:Q8T9F6_DROME	0.19	0.59	0.54	0.86	0.93	0.99
tr:E1JHB8:E1JHB8_DROME	0.2	0.6	0.54	0.86	0.93	0.99
tr:Q8T4M0:Q8T4M0_DROME	0.2	0.6	0.54	0.86	0.93	0.99
tr:Q7KTI7:Q7KTI7_DROME	0.2	0.59	0.54	0.86	0.93	0.99
tr:Q8T4L8:Q8T4L8_DROME	0.2	0.59	0.54	0.86	0.93	0.99
tr:Q9VLQ8:Q9VLQ8_DROME	0.2	0.59	0.54	0.86	0.93	0.99
tr:Q8IPG1:Q8IPG1_DROME	0.2	0.59	0.54	0.86	0.93	0.99
tr:Q7KTI8:Q7KTI8_DROME	0.2	0.59	0.54	0.86	0.93	0.99
tr:Q95P10:Q95P10_DROME	0.2	0.59	0.54	0.86	0.93	0.99
tr:E1JHB6:E1JHB6_DROME	0.19	0.56	0.54	0.86	0.93	0.99
tr:E1JHB7:E1JHB7_DROME	0.19	0.58	0.54	0.86	0.93	0.99

Pvr (FBgn0032006) associated phenotypes

decreased cell size, immune response defective, increased cell death, lethal - all die before end of pupal stage, mitotic cell cycle defective, neuroanatomy defective, partially, partially lethal - majority die, somatic clone, some die during pupal stage, temperature conditional, tumorigenic

(Information from FlyBase)
